# Transcriptome Analysis Revealing the Interaction of Abscisic Acid and Cell Wall Modifications during the Flower Opening and Closing Process of *Nymphaea lotus*

**DOI:** 10.3390/ijms232314524

**Published:** 2022-11-22

**Authors:** Weijuan Zhou, Zhaoji Li, Wentao Wu, Xia Zhao, Enbo Wang, Jian Wang, Xiqiang Song, Ying Zhao

**Affiliations:** Key Laboratory of Ministry of Education for Genetics and Germplasm Innovation of Tropical Special Trees and Ornamental Plants, Hainan Biological Key Laboratory for Germplasm Resources of Tropical Special Ornamental Plants, College of Forestry, Hainan University, Haikou 570228, China

**Keywords:** night-time-opening waterlily, gene regulation, flower open and closed, ABA, cell wall modification

## Abstract

As a tropical flower, *Nymphaea lotus* is a typical night-blooming waterlily used in water gardening. Its petals are rich in aromatic substances that can be used to extract essential oils and as flower tea. However, the short life of the flower seriously affects the development of its cut flowers. At present, neither the mechanism behind the night-opening waterlily flower’s opening and closing nor the difference between day-opening and night-opening waterlily flowers’ opening and closing mechanisms are clear. In this study, endogenous hormone contents of closed (CP) and open (OP) petals were measured, and transcriptome analysis of CP and OP petals was carried out to determine the signal transduction pathway and metabolic pathway that affect flower opening and closing. ABA and cell wall modification were selected as the most significant factors regulating flowering. We used qRT-PCR to identify the genes involved in the regulation of flower opening in waterlilies. Finally, by comparing the related pathways with those of the diurnal type, the obvious difference between them was found to be their hormonal regulation pathways. In conclusion, the endogenous ABA hormone may interact with the cell wall modification pathway to induce the flowering of *N. lotus*. Our data provide a new direction for the discovery of key factors regulating the flower opening and closing of *N. lotus* and provide basic theoretical guidance for future horticultural applications.

## 1. Introduction

*Nymphaea lotus* is a plant of *Nymphaea* in Nymphaeaceae. The *N. lotus*, also known as Egyptian white waterlily, is mainly distributed in tropical rainforests in Egypt, central and western Africa, Senegamnia, Guinea, Madagascar, and other places [1]. As the earliest branch of the main branch of the angiosperms phylogenetic tree, *Nymphaea* occupies an important position in evolution [2,3]. Besides its ornamental value, the waterlily also has edible and medicinal values [4,5]. The essential oil extracted from its petals has strong antioxidant activity [6] and significantly enriched flavonoids are present in the flower opening and closing process [7]. However, the short fresh-keeping period of cut flowers reduces their economic and ornamental value. Therefore, studying the movement of waterlily petals is helpful in trying to understand the movement characteristics of angiosperms and to improve the economic value of cut flowers.

Studies have shown that the opening and closing movement of petals involves the influence of factors of the external environment such as temperature, light, and humidity, alongside the role of endogenous rhythm, as well as the regulation of light signals and hormones, carbohydrates, and water transport [8,9]. Among these, hormones are crucial to the regulation of petal movement, and endogenous auxin, ethylene, gibberellin, and abscisic acid all have an effect on flower opening and closing [8]. Zhang et al. [3] have mentioned that auxin-related genes, such as GH3 and SAUR, can control circadian rhythm behind opening and closing of flowers. Ke et al. [10] found that auxin can regulate the movement of petals and control the opening and closing of waterlily flowers. Ethylene accelerates petal movement by promoting asymmetric growth at the base of petals [11]. Gibberellin promotes flowering [12] and abscisic acid is involved in controlling meristem function and flowering time [13]. Jasmonic acid from stamens regulates petal growth [14]. Cytokinins, on the other hand, slow down aging. Therefore, hormones interact with each other in the development and movement of petals and play complex and diverse roles. However, there have been some studies on the diurnal type of waterlily, but little is known about the nocturnal type of waterlily.

In waterlily petal movement, cell wall modification is essential for the changes in cell shape. For example, xyloglucan endotransglucosylase/hydrolase (XTH) and expansins (EXPs) can affect cell wall and petal movement by regulating differences [15,16,17]. In addition, cell wall modification is also related to hormones, and cell wall biosynthesis is considered to be a core event downstream of auxin regulation. Lehman et al. [18] have found that auxin-induced cell elongation and swelling is caused by modification of the cell wall. However, the depolymerization of pectin and xylan depends on the participation of ethylene [19]. MeJA promotes EXP and XTH expression and accumulation of EXP and XTH proteins in cut flower petals of Eustoma grandiflorum [20]. Gibberellin is involved in many plant development processes, including petal development, and can enhance cell wall invertase activity to participate in cell wall modification and promote petal cell expansion [21,22]. Abscisic acid can regulate the formation of the secondary cell wall and the accumulation of lignin, thus affecting the cell wall [23]. As for the effects of hormone and cell wall modification on the flowering motility rhythm of waterlilies, the effects of auxin and gibberellin on cell wall modification have been mentioned [10,24], effects which in turn affect the opening and closing of flowers. The mechanism of hormonal regulation and cell wall modification affecting the flowering of night-open waterlilies is not yet known, and the differences between the signal transduction and substance metabolism pathways affecting petal movement and day-open waterlilies are also poorly understood. 

In this study, *N. lotus* was selected as the research object. Through study of the pathways related to the opening and closing of the waterlily, including the regulation of light signals, hormone regulation, cell wall modification, water transport, and carbohydrate metabolism, it was found that the regulation of hormone and cell wall modification is particularly important for the regulation of petal movement. The hormone content in the petals of *N. lotus* during opening and closing of the flower was measured. Transcriptome analysis of petal samples at two time points associated with the opening and closing of the flower provides a comprehensive understanding of hormone synthesis and signal transduction, as well as the effect on the cell wall to regulate petal movement rhythm. By integrating the above two approaches, the hormones that significantly affect movement for the petals were analyzed. Finally, transcriptomic verification was carried out by qRT-PCR. This study provides a comprehensive perspective for the understanding of circadian rhythms in plants and offers useful information for studying the traits of angiosperms in flowering plants.

## 2. Results

### 2.1. Changes in Endogenous Hormone Content in N. lotus during Opening and Closing of the Flower

**Figure 1:**
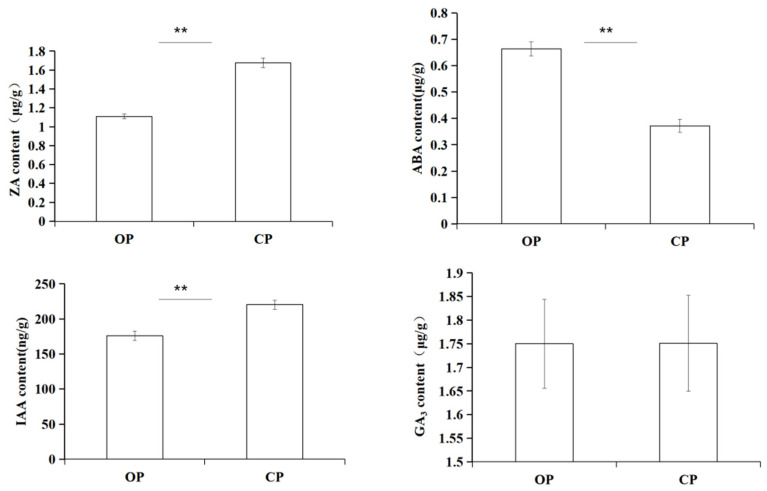
Content changes of ZA, ABA, IAA and GA_3_ hormones during OP and CP periods. ** Differences were considered extremely significant at *p* < 0.01.

The contents of zeatin (ZA) and auxin (IAA) in the CP group were significantly higher than those in the OP group, and the ABA content in the OP group was significantly higher than that in CP group. Thus, the ABA content increased from flower closure to opening. There was no significant difference in gibberellin (GA_3_) content (Figure 1).

### 2.2. Transcriptome Profiling of the Waterlily Flower at Open and Closed Periods

**Figure 2:**
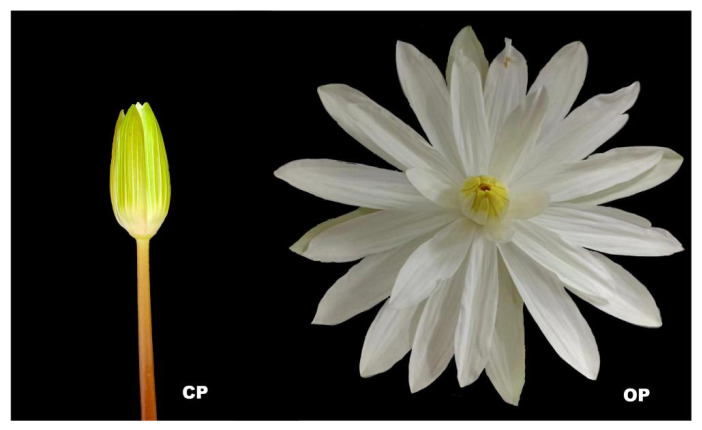
Picture of *N. lotus* closed (CP) and open (OP).

**Figure 3:**
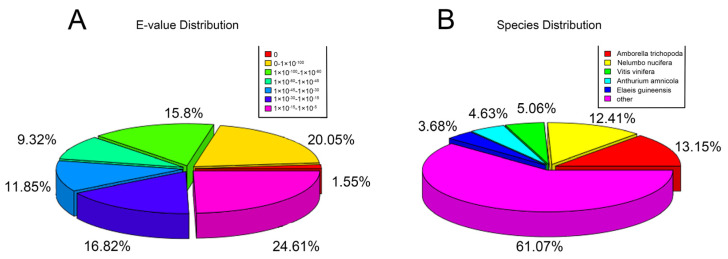
NCBI-NR annotations. (**A**) NCBI-NR annotated species distribution map. (**B**) E value distribution of NCBI-NR annotation.

To elucidate the mechanism of diurnal closing and night-opening petal movement in the *N. lotus*, we performed de novo RNA-Seq analysis on petals that opened and closed on the same day. Closed (CP) petals were the control, and open (OP) petals were the experimental group (Figure 2). After assembly and redundancy removal, 109,811 unigenes were obtained, among which the median length was 1455 nt and the value of N50 was 2410 nt. Unigene sequences were compared with protein databases, and 107,888, 81,947, and 83,072 annotations were obtained from the Nr database, KEGG database, and Swiss-Prot database, respectively. According to NCBI NR annotation and E-value distribution, 53.28% annotation sequences had strong homology (e-value < 10^−45^). According to NCBI NR notes, *Amborella trichopoda*, *Nelumbo nucifera*, *Vitis Vinifera*, *Anthurium amnicola*, and *Elaeis guineensis* all have mostly similar sequences (Figure 3). By studying gene expression differences during the open and closed periods, 2595 DEGs were identified by comparing the two periods, of which 783 were elevated and 1812 were decreased (Figure S1). According to GO and KEGG enrichment analysis, single-organism organelle tissue, biosynthesis, nitrogen compound metabolism, cell biosynthesis, negative regulation of cellular macromolecule biosynthesis, and pyruvate metabolism are the most significant GO enrichment terms in CP vs. OP. Carbon metabolism, protein output, and oxidative phosphorylation are the most representative KEGG pathways (Figures S2 and S3). These data suggest that negative regulation of multiple biosynthesis processes leads to the flower opening and closing of *N. lotus* at night and at day and that these flower opening and closing processes involve glucose metabolism, carbon metabolism, and oxidative phosphorylation. In order to further study the changes of gene expression in petal cells during movement of the petals, we investigated the important signaling pathways that affect flower development and movement for the petals, including hormone regulation and cell wall modification. Biosynthesis and signal transduction were integrated to analyze the mechanism of flower opening at night and closing at day.

#### 2.2.1. Gene Regulation Involved in Cell Signal Transduction of Waterlily Petals

- Ca^2+^ Signaling

**Figure 4:**
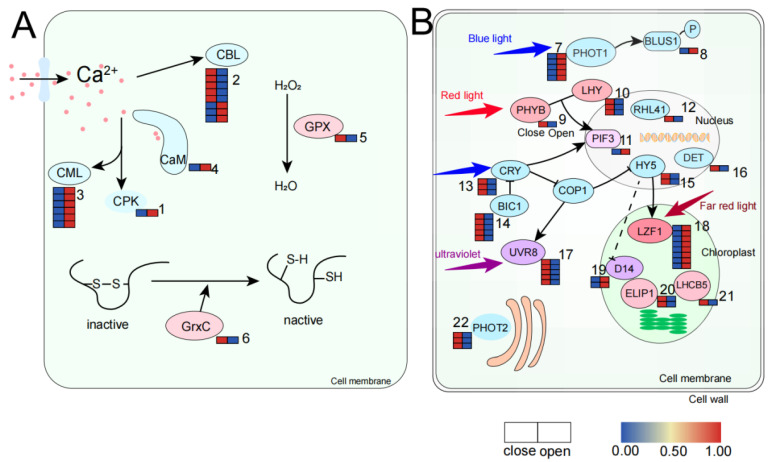
Expression patterns of genes in signal transduction pathways during open and closed periods. (**A**) Gene expression of Ca^2+^ signaling and ROS signaling pathway (the corresponding genes are listed in Table 1 according to the labeled number “1” to “6” in each map). (**B**) Gene expression in the photosignaling pathway (the corresponding genes are listed in Table 2 according to the labeled number “7” to “22” in each map). Red and blue colors indicate upregulated and downregulated transcripts, respectively. Proteins of the same color represent the fact that they are in the same signaling pathway. The blue color in (**A**) represents the Ca^2+^ signal transmission pathway, and the light pink color represents the ROS signal transmission pathway. In (**B**), blue represents the blue-light signal transmission pathway, pink represents the red-light signal transmission pathway, dark pink represents the far-red light signal transmission pathway, and purple represents the ultraviolet signal transmission pathway. The lilac in PIF3 represents the fact that the PIF3 gene is affected by both red and blue light.

Calcium signal transduction needs to be sensed and decoded by calcium ion sensing proteins including calmodulin (CMLs), calmodulin (CAM), calcium-dependent protein kinases (CPKs), and calciniurin B-like proteins (CBLs), which interact to form complex signaling networks. The downstream responses of calcium signals are mediated [25]. In transcriptome data, some genes of CML, CAM, CPK, and CBL were upregulated when opened (Figure 4A and Table 1).

- ROS Signaling

ROS is an unstable active molecule that is toxic to cells and regulating ROS homeostasis is essential to protect cells from dysfunction, senescence, and death. ROS can also regulate cell expansion through its effect on the cell wall. For example, heterogeneous hydrogen peroxide, hydroxyl radical, and superoxide can affect the hardness of the cell wall and thus affect the cell expansion rate [26,27]. In transcriptome data, glutathione peroxidase (GPX) and glutathione (GRX) were highly expressed in the closure phase (Figure 4A and Table 1).

- Light Signaling

Different light qualities can affect the opening and closing of the flower, and the genes related to light signaling are regulated differently in the flower opening and closing period of *N. lotus*. In previous studies, it has been shown that the key receptors for sensing light are plant pigments (PHYs), blue light receptor-cryptochrome (CRY), photophysin (PHOT), ultraviolet receptor 8 (UVR8), and other downstream response factors including photosensitive pigment interaction factor (PIF3), constitutive photomorphogenic 1 (COP1), elongated hypocotyl 5 (HY5), etc. The coordination of these light signaling factors affects various aspects of plant development, including flowering rhythm [28]. PHOT1 was highly expressed after blue light signal induction during the OP period. LZF1 was highly expressed during OP induced by far-red light signaling. The receptors of red light and ultraviolet light, PHYB and UVR8, were highly expressed in the CP stage, and cryptochrome (CRY) was also low in the OP stage. RHL41 was highly expressed after red or blue light signal induction during the CP stage. DET1 positively regulates the degradation of HY5 protein and is thus highly expressed in the CP stage, just as HY5 is (Figure 4B and Table 2).

- Hormone Coordination

**Figure 5:**
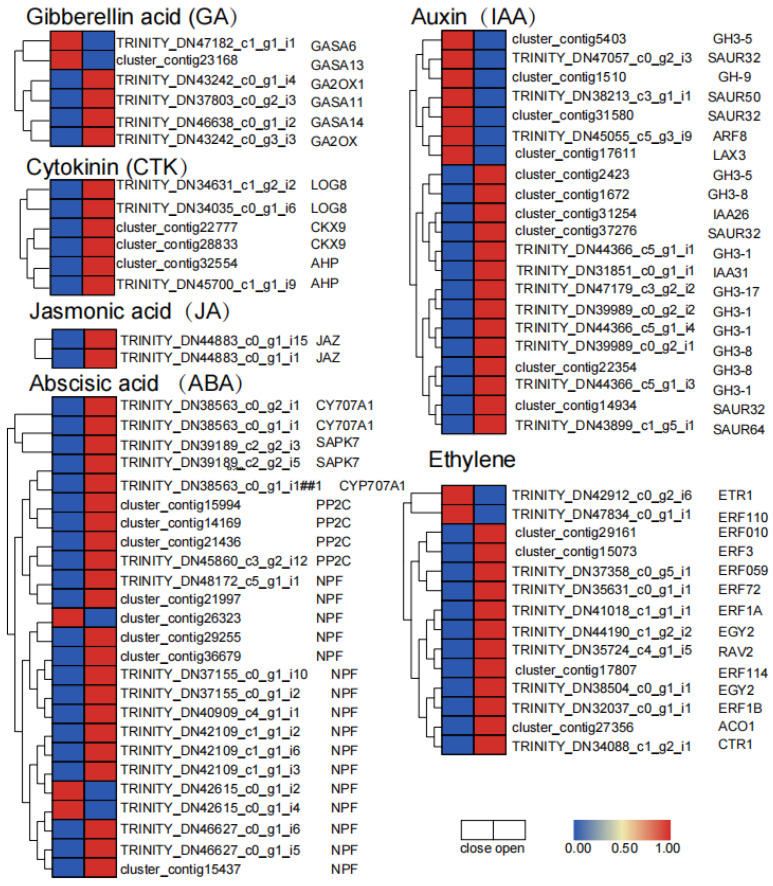
Heat map of gene expression in the CP and OP periods of hormone signaling pathways.

The plant hormone auxin plays a very important regulatory role in plant growth and development. It can induce the expression of a series of auxin early response genes, mainly including SAURs, AUX/IAAs, and Gretchen Hagen3s (GH3) gene families. Auxin signal transduction and synthesis regulate the flower opening and closing of *N. lotus*. The SAUR gene family is the largest family of auxin response factors unique to plants, are able to respond to early auxin induction and are capable of being regulated by circadian rhythm, and then in turn regulating cell expansion, leaf senescence, and other plant development processes [29]. In transcriptome data, SAUR was upregulated in both open and closed periods, and SAUR50 was upregulated in the CP period. SAUR64 was upregulated during OP. In transcriptome data, both IAA31 and IAA26 were upregulated at the OP stage, and the auxin response factor ARF8 protein was upregulated at the CP stage. GH3.5 and GH9 were upregulated in the CP stage, and GH3.5, GH3.8, GH3.1, and GH3.17 were upregulated in the OP stage, suggesting that different GH3 family members played different roles in flower opening and closing stages (Figure 5).

The JAZ protein in the jasmonic acid (JA) signaling pathway is a zinc finger protein located in the nucleus and plays an important role in the response to the JA signaling pathway. As a common receptor of JA-lle, and alongside COI1, it responds to JA signals and inhibits the activity of downstream transcription factors (TF), thus playing a role in negatively regulating both ABA-inhibiting seed germination and ABA-responsive gene expression [30]. In the transcriptome data, the JAZ gene was up-regulated during OP (Figure 5).

Gibberelcin (GA) can promote plant cell elongation. In transcriptome data, GASA6 and GASA13 were upregulated at the CP stage, indicating that GA_3_ and DELLA were accumulated at closure stage, and GA2ox, GASA11, and GASA14 were upregulated at the OP stage (Figure 5).

Cytokinin (CTK) plays an important regulatory role in plant meristem and morphogenesis, and cytokinin signaling has been reported to be involved in the regulation of early flowering [31]. In transcriptome data, the cytokinin synthesis gene LOG8 was upregulated during OP, as was the cytokinin degradation enzyme CKX, which maintains optimal levels of cytokinin in plants for growth. The cytokinin sensor phosphoric acid transfer protein AHP, containing histidine, was also upregulated during OP (Figure 5).

Abscisic acid (ABA) is involved in the regulation of plant growth and development. In the transcriptome data, CYP707A1, SAPK7, and PP2C were all upregulated in the open period, while NPF was upregulated in both the open and closed periods, but most of these were upregulated in the open period (Figure 5).

Ethylene is an important gaseous plant hormone associated with many physiological effects in plant growth and development. In transcriptomic data, ETR1 and ERF110 were upregulated during the closure period, while ERF010, ERF3, ERF059, ERF72, ERF1A, EGY2, RAV2/ERF, ERF114, ERF1B, ACO1, and CTR1 were upregulated during OP. These results indicate that ethylene synthesis and signal transduction are stimulated during the opening process of waterlilies (Figure 5).

#### 2.2.2. Gene Regulation Involved in the Cell Metabolism of Waterlily Petals

- Water Transportation

**Figure 6:**
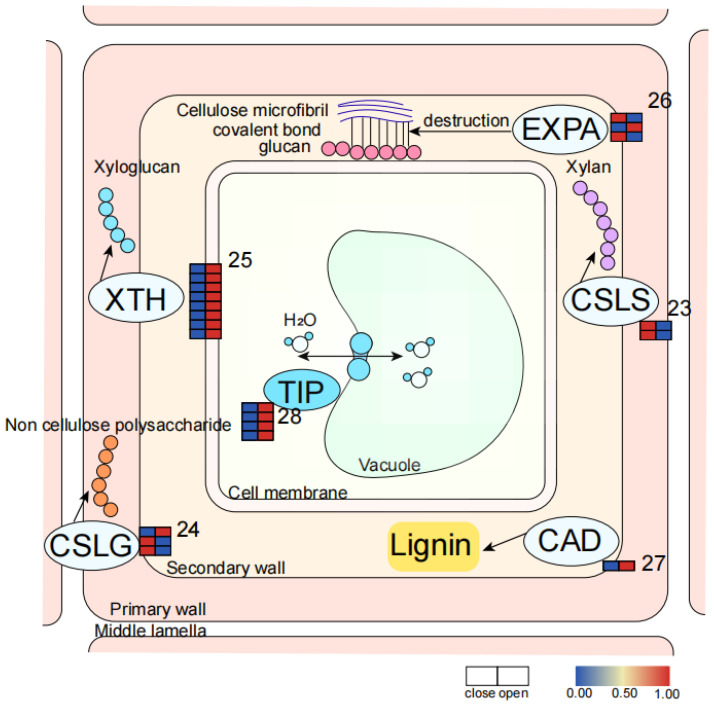
Expression pattern diagram of genes of the water channel pathway and cell wall modification pathway (the corresponding genes are listed in Table 3 according to the labeled number “23” to “27” in each map). Red and blue colors indicate upregulated and downregulated transcripts, respectively. Proteins of the same color represent the fact that they are in the same signaling pathway. Blue represents water transport pathways. White represents the cell wall modification pathway.

Cell swelling is the cause of the opening and closing of waterlily petals, and cell swelling is closely related to water transportation [32]. Aquaporins (APQs) control intracellular water transport and are involved in many developmental processes in plants [33]. In the transcriptome data, TIP was highly expressed at the OP stage, and vacuolar dilation led to cell expansion and flower opening (Figure 6 and Table 3).

- Cell Wall Modification

Opening and closing of the flower may be due to reversible cell expansion and contraction [9], during which cell wall modification is inevitable. The cell wall is composed of an intercellular layer, a primary wall, and a secondary wall. The intercellular layer is mainly pectin; the primary wall is mainly structural proteins, such as cellulose, and hemicellulose; and the secondary wall is mainly cellulose and lignin [34]. In the transcriptome data, XTH was highly expressed during OP. CSLG is a β-glycan synthase, most of which is highly expressed in the CP stage. CSLS is a xylan glycosyltransferase that is highly expressed in the CP stage. EXPA is an expansible protein that is highly expressed in the CP stage, and some of it is also highly expressed in the OP stage. CAD is involved in the lignin biosynthesis and phenylpropane biosynthesis pathway [35] and is highly expressed in the OP period (Figure 6 and Table 3).

### 2.3. qRT-PCR Analysis

In order to verify the effects of hormones on the cell wall and the credibility of transcriptomic sequencing data, primers were designed for fluorescence quantitative analysis of qRT-PCR sequences of 11 unigenes, and the qRT-PCR results of nine unigenes were consistent with RNA-Seq results (Table 4). However, the regulation of three unigenes was contrary to the sequencing results.

## 3. Discussion

Plants bloom for reproduction. Whether they bloom during the day or at night, rhythmically and repeatedly or only once, they are all designed to spread divisions and fertilize pistils to the maximum extent possible under the current environmental conditions [9]. Waterlilies are insect-pollinated plants. Most waterlilies that bloom at night and close in the day are tropical waterlilies, which may be to avoid the threat of high temperature during the day and cater to the activity period of insects so that the plants can have better reproduction [36]. Some factors, including hormone regulation, water transport, cell wall modification, Ca^2+^ signaling pathway, ROS signaling pathway, and light signaling pathway, participate in the regulation of the opening and closing processes of the flower. Transcriptional analysis of *N. lotus* showed that hormones have a great influence on the cell wall, thus affecting the time of flower opening. The above pathways were also discussed in our laboratory’s previous studies on *N*. ‘Blue Bird’ that open during the day (T1) and close at night (T2) [24]. However, the differences and similarities between the waterlilies that open at night and those that open during the day in terms of the above pathways are not known at present. We discussed the related mechanisms of flower opening and closing of *N. lotus* and compared the similarities and differences between the regulation mechanisms of night opening and day closing and day opening and night closing, and preliminarily explored the key genes regulating these two phenomena. The mechanism of the interaction between hormones and cell wall modification on the flower opening and closing of waterlily was analyzed by the application of hormones.

### 3.1. Similarities and Differences in Flower Opening and Closing Mechanism between N. ‘Blue Bird’ and N. lotus

*N*. ‘Blue Bird’, a tropical waterlily variety that opens during the day, and *N. Lotus,* a tropical waterlily variety that opens at night, are implicated in open–closed mechanism research, and no research has yet elucidated the similarities and differences between their hormonal regulation, water transport, cell wall modification, Ca^2+^ signaling pathways, ROS signaling pathways, and light signaling pathways in the several pathways that have been analyzed.

**Figure 7:**
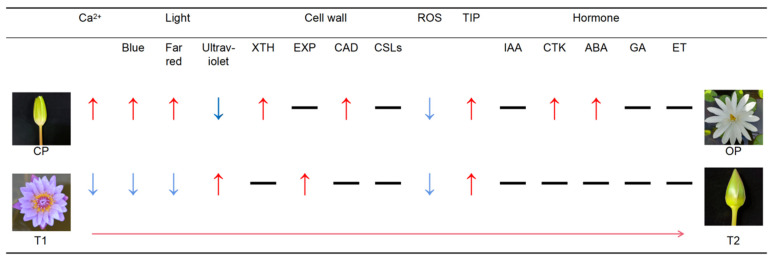
Schematic model of the molecular mechanism differences in petal opening and closing between two waterlily species. ↑ indicates upregulated gene expression, ↓ downregulated gene expression, — indicates that these genes jointly regulate the opening and closing of petals, ⟶ indicates the process from CP to OP and the process from T1 to T2.

CML, CBL, and other related genes in the Ca^2+^ signaling pathway of *N. lotus* were highly expressed in the opening period, and there was no significant difference between *N. lotus* and *N*. ‘Blue Bird’. The light signal pathway, *N. Lotus,* and *N*. ‘Blue Bird’ are regulated by blue, red/far-red, and ultraviolet light. Both blue and far-red light regulate the opening of the flowers of the two types of waterlilies. Ultraviolet light regulates the closure of the flowers of both types of waterlilies. Therefore, there is no significant difference in the regulation of the two types of waterlilies by the light signaling pathway. In terms of cell wall modification pathways, both night- and day-opening waterlilies have similar genes that regulate cell wall remodeling, and which are involved in cellulose synthesis when open [16] and depend on cell wall acidification and cell wall microfilament rearrangement when closed [17] (Figure 7).

However, in the ROS signaling pathway of *N. lotus*, glutathione peroxidase (GPX) and glutathione (GRX) were highly expressed at the closure stage, which is contrary to that of *N*. ‘Blue Bird’, but the ROS pathway was mainly involved in petal senescence in *N*. ‘Blue Bird’, so the ROS pathway may be involved in inducing petal closure of *N. lotus*. TIP was highly expressed in the OP and T2 stages, indicating that the water transport activity period of night and day waterlily vacuoles was different. In the day-opening waterlily, NIPs and PIPs are highly expressed in the T2 period, meaning that the daytime-opening waterlily transports moisture more frequently between adjacent cells and the night-opening waterlily transports moisture more frequently to its vacuole, rising in the night air humidity and also making a water gap from the outside world, wherein the energy is used for the transport of water and material in the cell (Figure 7). 

In the pathway of hormone regulation, genes related to auxin IAA, gibberellin GA, abscisic acid ABA, and ethylene were highly expressed at OP, CP, and T1 and T2 stages. The auxin response factor ARF8 was highly expressed at the CP stage in the IAA pathway of night-opening waterlilies. This indicates that ARF8 may control the level of free IAA in the form of negative feedback [37], which inhibits the synthesis of IAA, indicating that there is negative feedback regulation during closure. The homologous sequence of cytokinin synthesis gene LOG and cytokinin degradation enzyme CKX in CTK were highly expressed in OP and T2 stages, suggesting that CTK regulates flower opening in the night type and flower closure in the day type. JAZ in JA is highly expressed during OP, and JAZ is a negative regulator that blocks the initiation of JA-responsive transcription factors [38]. JMT and JAR1 homologous sequences were highly expressed at the T1 stage, suggesting that jasmonic acid had little effect on the opening of nocturnal waterlilies but was involved in regulating the opening of diurnal waterlilies. In the GA pathway, the DELLA protein plays an important role in GA signal transduction, and DELLA protein degradation is considered to be a marker of GA growth promotion and other GA responses [39]. GASA6 is located downstream of the DELLA protein and is inhibited by DELLA. The expression of GASA13 is induced by GA3, and the expression of GASA11 is induced by exogenous GA3, but GA3 has no significant effect on the expression of GASA14 [40]. GA2ox can catalyze the conversion of bioactive GA to an inactive form [41]. Different from diurnal waterlilies, GASA11 and GASA14 are highly expressed in the OP period, which may regulate the flowering process of nocturnal waterlilies. In the ABA pathway, the CYP707A1 protein participates in the oxidative degradation of abscisic acid [42], and the abscisic acid-activated protein kinase SAPK7 may be induced by abscisic acid [43]. PP2C is one of the centers of the ABA signal transduction pathway ABA–PyR1–PP2C complex [44], and NPF is involved in abscisic acid transport and mediates ABA into cells [45]. Transcriptomic data suggest that ABA regulates the flowering of both night and day waterlilies. In the ethylene pathway, ETR1 and CTR1 are negative regulators of ethylene signal transduction [46]. ERF is an ethylene response factor and is expressed by ethylene [47]. Studies have shown that inhibition of slEGY2 gene expression increases endogenous ethylene content in tomato seedlings, and slEGY2 may be an inhibitor of ethylene synthesis degradation [48]. ACO catalyzes the conversion of ACC to ethylene [49]. Negative regulator CTR1 was highly expressed at the OP stage, and ethylene receptor ETR1 was highly expressed at the CP stage. Other genes were similar to those of the diurnal waterlily, suggesting that the opening of nocturnal waterlilies is negatively regulated by genes related to the ethylene pathway (Figure 7).

It can be seen from the above results that hormone regulation induces the opening of the two types of waterlilies in significantly different ways, and hormones also affect the cell wall modification. In combination with the determination results of hormone content in the early stage, it is concluded that the ABA hormone induces the opening of the flower of *N. lotus*.

### 3.2. The Interaction between ABA Hormone and Cell Wall Modification Affected the Opening of N. lotus

Through the determination of endogenous hormones in the petals of *N. lotus* in CP and OP periods, it can be seen from Figure 1 that the content of ABA in the OP period was significantly higher than that in the CP stage. Because waterlilies in the open period are collected from the closed to open period, ABA may be the most influential hormone in regulating the flowering of *N. lotus*. Results in the transcriptome data also show that ABA related genes *CY707A1*, *SAPK7*, *PP2C*, and *NPF* were highly expressed during the opening period (Figure 5). The flowering process involves cell wall modification, and XTH and CAD genes in the cell wall modification pathway are highly expressed during flowering. On the basis of the results of endogenous hormone content measurement and transcriptome analysis, we hypothesized that abscisic acid could induce the expression of genes related to cell wall modification. Therefore, low concentrations of abscisic acid may induce early flowering of waterlilies, which is consistent with the results of Campbell [50] and Liu [23].

## 4. Materials and Methods

### 4.1. Plant Materials and Growth Conditions

*N. lotus* was planted in Rongfeng Company base, Haikou city, Hainan province. Materials of *N. lotus* with the same openness at the same time were collected and placed in the laboratory of Hainan University (20°3 ‘31.31’ N, 110°19 ‘9.82’ E) for hydroponic growth at 25 °C, with light from 8:00 to 23:00 and darkness from 23:00 to 8:00.

### 4.2. Determination of Endogenous Hormone Content during the Flower Opening and Closing Process of N. lotus

The petals of *N. lotus* at the time of full closure (14:00) and at the time of full opening on the same day (20:00) were collected. Each biological replicate took 1 flower from each of the 10 plants of N. lotus (in the same period). The petals of each flower were taken 0.1 g according to the inner, middle and outer layers, for a total of 3 g. The samples were mixed and frozen milled and 0.2 g was taken as a biological replicate. There were 3 biological repeats in total. Frozen samples were ground to fine powders with a grinding machine (30 Hz, 1 min). The 0.2 g of petal powder were weighed and dissolved in a 0.5–1 mL extract solution containing methanol, water, and formic acid (*v*/*v*/*v* = 15:4:1). After 10 min of extraction, the supernatant was obtained by centrifugation for 5 min at 14,000 RPM. The extraction and centrifugation steps of all supernatants were combined and candied at 35 °C. The extracts were then resuspended with 100 μL of an 80% methanol–water solution and sonicated for 1 min, followed by heavy filtration through a 0.22-micron polytetrafluoroethylene membrane.

IAA, CTK, ABA, and GA3 were detected by enzyme-linked immunosorbent assay (ELISA), following the manufacturer’s instructions (Jingmei Biotechnology, Jiangsu, China). JA was analyzed by ultra-high-performance liquid chromatography (UPLC) (Shim-pack UFLC SHIMADZU CBM3OA, http//www.shimadzu.com.cn (accessed on 30 May 2021)) and a tandem mass spectrometry (MS/MS) (Applied Biosystems 6500 Quadrupole Trap, http://www.appliedbiosystems.com.cn/ (accessed on 30 May 2021)). The detailed procedures that were followed are described in [51]. The hormonal contents obtained in the analysis were expressed as pg or µg per gram fresh weight biological replicates for each time point.

### 4.3. RNA-Seq, Expression Annotation, GO and KEGG Pathway Enrichment and Gene Expression Patterns

Petal samples were collected at two time periods: closed group samples were collected at 14:00 and the open group samples were taken at 20:00 on the same day. Each biological replicate took 1 flower from each of the 10 plants of N. lotus (in the same period). The petals of each flower were taken 0.1 g according to the inner, middle and outer layers, for a total of 3 g. The samples were mixed and frozen milled and 0.6 g was taken as a biological replicate. There were 3 biological repeats in total. All collected petal samples were immediately frozen in liquid nitrogen and stored at −80 °C for RNA extraction. Six RNA-Seq libraries were constructed and sequenced on the IlluminaHiSeq^TM^2000 platform, and the obtained original data were removed with connectors and low-quality reads, which were then assembled ab initio using Triniyty (V2.4.0). Then, Tgicl (V5.18.2) was used to remove redundancy and further splice it. Finally, BLAST (2.7.1+) was used to conduct comparative filtering with the NR-plant library to obtain the final unigene. Blastx comparison was used to annotate these genes into the protein database Nr, Swiss-Prot, KEGG, and COG (E-value < 0.00001), and the difference in single gene expression abundance was expressed by FPKM. FPKM was the number of reads per 1K base from map to exon in reads on 1 million map. Kallisto (0.43.1) was used for differential expression analysis, and edgeR (3.16.5) was used to obtain the false discovery rate (FDR), a statistical method to correct *p*-values in multiple test correction. Differentially expressed genes were defined as those with FDR ≤ 0.001 and more than 2 instances of difference. Pathway analysis of DEGs was performed by KEGG. ClusterProfiler was used for gene function enrichment (v3.3.0). On the basis of the hypergeometric test, significantly enriched GO annotations and KEGG pathways were identified (q-value ≤ 0.05). GO annotation reference site: http://www.geneontology.org/ (accessed on 28 January 2022); KEGG annotation reference site: http://www.genome.jp/kegg/ (accessed on 28 January 2022).

### 4.4. qRT-PCR Assay

In order to verify the results of RNA-Seq, 13 unigenes were selected for real-time fluorescence quantitative PCR (qRT-PCR) analysis, and the primer sequences designed by NCBI were cited as shown in Supplementary Table S1. Actin11 was selected as the internal reference [24]. The real-time ReLRT PCR experiment was carried out on the instrument of Applied Biosystems Quantum Studio 3 and 5 System (Thermo Fisher Scientific, Waltham, MA, USA). SYBRGREEN super mixture (Applied Biological System) was used, and each sample was repeated 3 times.

### 4.5. Statistical Analysis

Differences between samples were tested for statistical significance using the Student’s *t*-test method as implemented by Excel software (2021). Fisher’s least significant difference (LSD) multiple comparisons were used for statistical analyses at the confidence level of 95%.

## 5. Conclusions

Through the determination of endogenous hormone content, it can be seen that the endogenous hormone ABA plays an important role in the opening process of *N. lotus*. By transcriptome analysis of the signal transduction pathways and metabolic pathways associated with flower opening and closure, important factors regulating flowering in ABA and cell wall modifications were uncovered. Comparing the relevant signaling pathways related to flower opening and closing of nocturnal and diurnally opening waterlilies, it can be seen that the pathway of hormones regulating flower opening and closing in the two types of waterlilies was obviously different. Combining the above results, we find that low concentrations of ABA hormone may induce *N. lotus* flower to open early by interacting with cell wall modifications. This study provides useful information for the study of the traits of flowering plants in angiosperms.

## Figures and Tables

**Table 1:** Ca^2+^ signaling and ROS signaling pathway gene expression levels (corresponding to Figure 4A).

Heatmap Number	Gene ID	Best Homolog	FPKM (Close)	FPKM (Open)
1	TRINITY_DN33954_c0_g1_i1	CPK20	24.67	222.67
2	TRINITY_DN43164_c0_g1_i7	CBL21	103.08	5.62
	cluster_contig14104	CBL3	76.85	5.33
	TRINITY_DN43164_c0_g1_i6	CBL23	176.16	16.00
	cluster_contig11726	CBL23	193.56	21.18
	TRINITY_DN42937_c3_g3_i8	CBL12	1537.17	235.54
	TRINITY_DN34147_c0_g1_i1	CBL5	30.33	191.67
	cluster_contig33272	CBL12	76.41	510.81
	cluster_contig33312	CBL12	28.88	238.84
3	TRINITY_DN37281_c0_g1_i1	CML49	25.82	96.47
	TRINITY_DN43916_c0_g1_i2	CML38	104.05	405.90
	cluster_contig32852	CML50	29.45	127.22
	cluster_contig17156	CML11	13.06	80.58
	TRINITY_DN43916_c0_g1_i1	CML38	84.95	503.44
	TRINITY_DN30645_c0_g1_i1	CML3	9.33	70.00
4	TRINITY_DN33584_c4_g1_i6	CAM	6.04	59.63
5	TRINITY_DN35826_c0_g2_i1	GPX	246.73	31.54
6	TRINITY_DN38584_c2_g1_i3	GrxC	179.65	31.69

**Table 2:** Expression levels of light-signal-related genes (corresponding to Figure 4B).

Heatmap Number	Gene ID	Best Homolog	FPKM (Close)	FPKM (Open)
7	cluster_contig33844	PHOT1	136.62	576.57
	TRINITY_DN46984_c2_g2_i1	Phot1	10.07	60.37
	TRINITY_DN46984_c2_g2_i3	PHOT1	98.18	421.90
	cluster_contig4683	Phot1	0.00	35.67
	TRINITY_DN39051_c0_g1_i1	PHOT1	35.71	146.36
8	cluster_contig24741	BLUS1	130.88	566.19
9	TRINITY_DN39859_c1_g2_i5	PHYB	2037.26	369.42
10	cluster_contig16390	LHY	9279.65	1215.09
	TRINITY_DN43711_c0_g2_i1	LHY	2875.53	464.13
	TRINITY_DN43711_c0_g2_i5	LHY	15,407.33	2664.26
11	TRINITY_DN45082_c1_g1_i9	PIF3	97.68	485.99
12	TRINITY_DN40718_c0_g1_i2	RHL41	2465.50	232.00
13	cluster_contig33117	CRY1	371.33	55.86
	TRINITY_DN46110_c0_g1_i11	CRY1	90.27	17.33
	cluster_contig19944	CRY1	241.05	52.90
14	TRINITY_DN46446_c3_g1_i8	BIC1	7182.97	286.44
	cluster_contig37221	BIC1	2300.67	97.00
	TRINITY_DN46446_c3_g1_i6	BIC1	144.56	10.94
	TRINITY_DN46446_c3_g1_i1	BIC1	842.99	188.47
	cluster_contig4395	BIC1	37.35	0.00
15	TRINITY_DN36278_c0_g1_i4	HY5	534.41	107.30
	TRINITY_DN36278_c0_g1_i1	HY5	179.11	29.05
16	cluster_contig33432	DET1	102.44	20.60
17	cluster_contig20006	UVR8	166.67	12.87
	TRINITY_DN41117_c2_g1_i3	UVR8	127.55	11.00
	TRINITY_DN41521_c1_g1_i5	UVR8	1277.37	284.49
	TRINITY_DN48407_c3_g1_i15	UVR8	2526.64	229.06
	cluster_contig7928	UVR8	3819.14	796.44
18	cluster_contig31250	LZF1	158.74	721.23
	TRINITY_DN37789_c5_g1_i1	LZF1	74.08	521.48
	TRINITY_DN47837_c0_g1_i8	LZF1	7.85	288.19
	cluster_contig27900	LZF1	5.16	202.30
	TRINITY_DN47837_c0_g1_i6	LZF1	7.93	423.05
	cluster_contig23776	LZF1	10.60	566.78
	cluster_contig20252	LZF1	2.79	173.56
	TRINITY_DN47837_c0_g1_i7	LZF1	0.00	28.78
19	cluster_contig21245	D14	3.75	44.71
	TRINITY_DN44313_c1_g1_i3	D14	1.73	83.11
20	TRINITY_DN32565_c1_g2_i1	ELIP1	14,497.87	101.38
	TRINITY_DN32565_c1_g2_i3	ELIP1	33,519.13	564.96
21	TRINITY_DN45074_c2_g1_i5	LHCB5	827.38	156.19
22	TRINITY_DN44026_c1_g1_i23	PHOT2	112.08	11.49
	TRINITY_DN44026_c1_g1_i7	PHOT2	202.36	5.67
	TRINITY_DN44026_c1_g1_i1	PHOT2	94.08	0.00

**Table 3:** Expression levels of genes related to cell wall modification.

Heatmap Number	Gene ID	Best Homolog	FPKM (Close)	FPKM (Open)
23	cluster_contig16162	CslC8	190.47	26.60
	cluster_contig34906	CslC4	136.02	19.46
24	TRINITY_DN43633_c1_g2_i1	CslG3	1335.50	8318.64
	TRINITY_DN44782_c0_g1_i6	CslG2	5551.84	684.41
	cluster_contig12214	CslG2	538.98	32.21
25	TRINITY_DN40931_c0_g2_i2	XTH-15	83.94	5911.52
	TRINITY_DN41938_c0_g1_i4	XTH-23	64.76	496.04
	TRINITY_DN43609_c2_g1_i1	XTH-3	33.90	558.46
	TRINITY_DN43609_c2_g1_i8	XTH-3	56.87	566.53
	TRINITY_DN47961_c4_g1_i6	XTH-2	378.57	1794.97
	cluster_contig5240	XTH-2	179.43	2105.00
	cluster_contig32082	XTH-13	9.67	188.96
	TRINITY_DN33636_c9_g2_i3	XTH-9	5.86	124.60
26	TRINITY_DN32754_c1_g1_i2	EXPA10	280.11	39.51
	TRINITY_DN32754_c2_g2_i1	EXPA1	19.67	123.33
	TRINITY_DN32754_c3_g2_i2	EXPA1	450.23	90.96
27	TRINITY_DN36767_c0_g2_i3	CAD	519.16	3871.91

**Table 4:** The qRT-PCR with the differential expressed genes.

Comparison	MgPUTs ID	RT-PCR2^−ΔΔCT^	*p*-Value	Consistency with RNAseq	Annotation (Blastx)
OP vs. CP	cluster_contig33312	16.64 ± 2.64	2.04 × 10^−13^	Y	CBL-interacting serine/threonine-protein kinase 12
	TRINITY_DN43916_c0_g1_i1	59.44 ± 5.15	3.90 × 10^−5^	Y	Calcium-binding protein CML38
	TRINITY_DN38584_c2_g1_i3	3.00 ± 1.46	2.52 × 10^−5^	N	Glutaredoxin-C4
	TRINITY_DN46984_c2_g2_i3	21.33 ± 2.56	1.85 × 10^−12^	Y	Phototropin-1
	cluster_contig37221	0.41 ± 0.06	4.77 × 10^−23^	N	Protein BIC1
	TRINITY_DN45074_c2_g1_i5	46.21 ± 0.57	8.58 × 10^−16^	N	Light-harvesting complex II protein 5
	cluster_contig32082	1.43 ± 0.17	1.54 × 10^−11^	Y	Putative xyloglucan endotransglucosylase/hydrolase protein 13
	cluster_contig15073	0.84 ± 0.13	5.22 × 10^−10^	Y	Probable zinc metalloprotease EGY2
	TRINITY_DN44883_c0_g1_i1	17.00 ± 3.51	1.16 × 10^−16^	Y	Jasmonate ZIM domain-containing protein 1
	cluster_contig22354	19.61 ± 4.01	3.68 × 10^−11^	Y	Auxin-responsive GH3-like protein 8
	cluster_contig15994	66.56 ± 25.29	9.86 × 10^−11^	Y	Abscisic acid 8’-hydroxylase 1

## Data Availability

The datasets presented in this study can be found in online repositories. The names of the repository/repositories and accession number(s) can be found below: https://www.editorialmanager.com/indcro/l.asp?i=913920&l=X4TY8EBP (accessed on 14 August 2022).

## Supplementary Materials

The following supporting information can be downloaded at: https://www.mdpi.com/article/10.3390/ijms232314524/s1.
