# Supplementary material for: Transcriptome Analysis Revealing the Interaction of Abscisic Acid and Cell Wall Modifications during the Flower Opening and Closing Process of Nymphaea lotus
Source: Int J Mol Sci. 2022 Nov 22;23(23):14524. doi: 10.3390/ijms232314524 (PMC9740110; doi:10.3390/ijms232314524)
Supplement: Supplementary file 1 [file ijms-23-14524-s001.zip › ijms-1941285-supplementary.pdf]

Figure S1 Statistics of differentially expressed genes.

Figure S2 GO enrichment analysis.

Figure S3 KEGG enrichment analysis.

Table S1 Primer sequences for qRT-PCR.

Figure S1

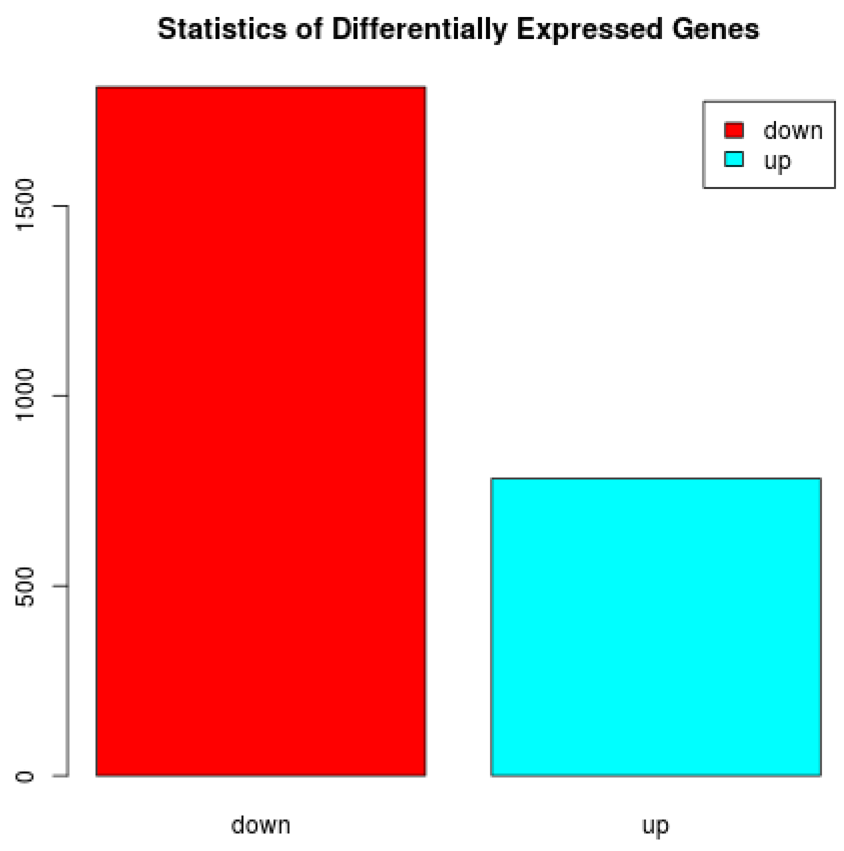
Statistics of Differentially Expressed Genes
down
up
0
500
1000
1500
down
up

Figure S2

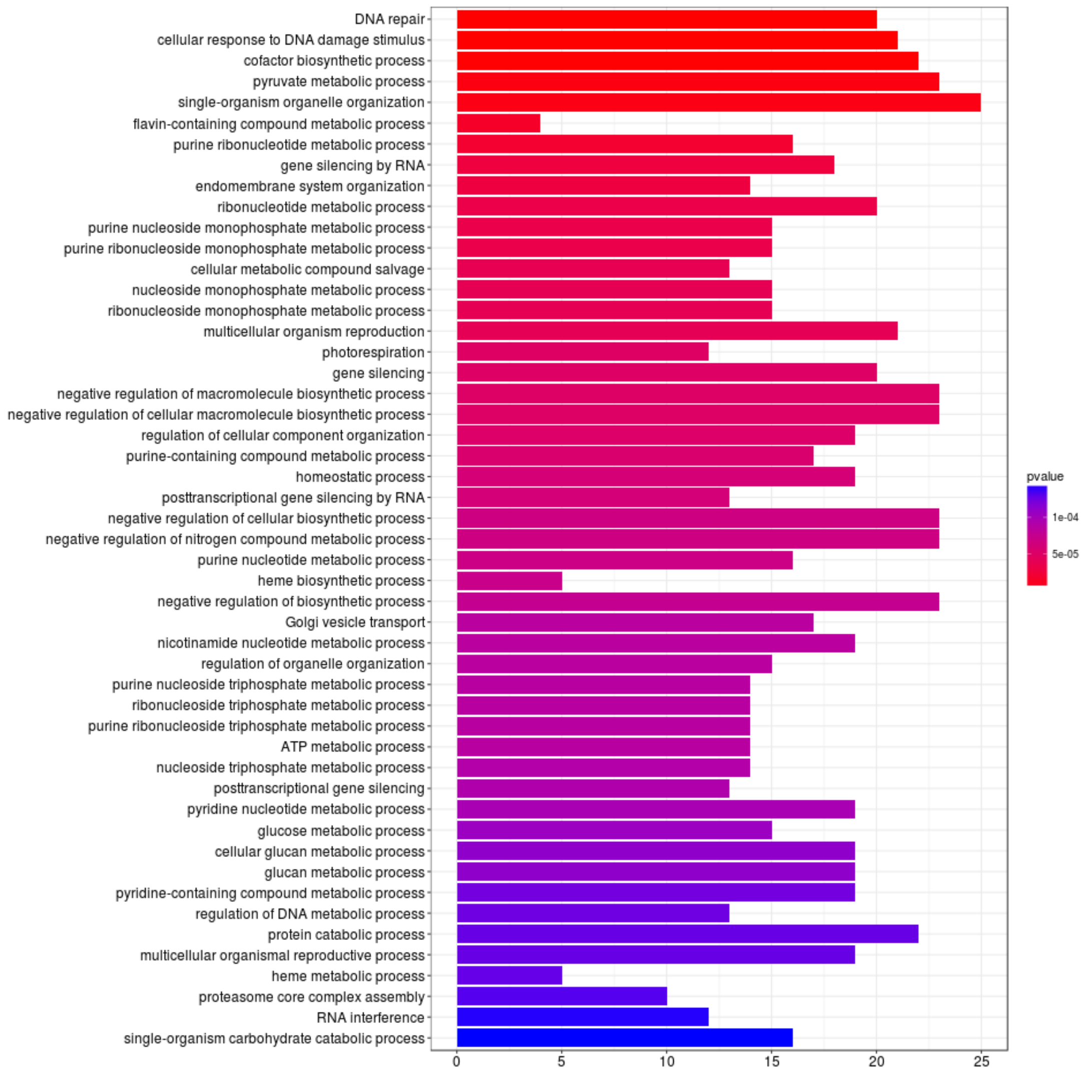
DNA repair
cellular response to DNA damage stimulus
cofactor biosynthetic process
pyruvate metabolic process
single-organism organelle organization
flavin-containing compound metabolic process
purine ribonucleotide metabolic process
gene silencing by RNA
endomembrane system organization
ribonucleotide metabolic process
purine nucleoside monophosphate metabolic process
purine ribonucleoside monophosphate metabolic process
cellular metabolic compound salvage
nucleoside monophosphate metabolic process
ribonucleoside monophosphate metabolic process
multicellular organism reproduction
photorespiration
gene silencing
negative regulation of macromolecule biosynthetic process
negative regulation of cellular macromolecule biosynthetic process
regulation of cellular component organization
purine-containing compound metabolic process
homeostatic process
posttranscriptional gene silencing by RNA
negative regulation of cellular biosynthetic process
negative regulation of nitrogen compound metabolic process
purine nucleotide metabolic process
heme biosynthetic process
negative regulation of biosynthetic process
Golgi vesicle transport
nicotinamide nucleotide metabolic process
regulation of organelle organization
purine nucleoside triphosphate metabolic process
ribonucleoside triphosphate metabolic process
purine ribonucleoside triphosphate metabolic process
ATP metabolic process
nucleoside triphosphate metabolic process
posttranscriptional gene silencing
pyridine nucleotide metabolic process
glucose metabolic process
cellular glucan metabolic process
glucan metabolic process
pyridine-containing compound metabolic process
regulation of DNA metabolic process
protein catabolic process
multicellular organismal reproductive process
heme metabolic process
proteasome core complex assembly
RNA interference
single-organism carbohydrate catabolic process
0
5
10
15
20
25
pvalue
1e-04
5e-05

Figure S3

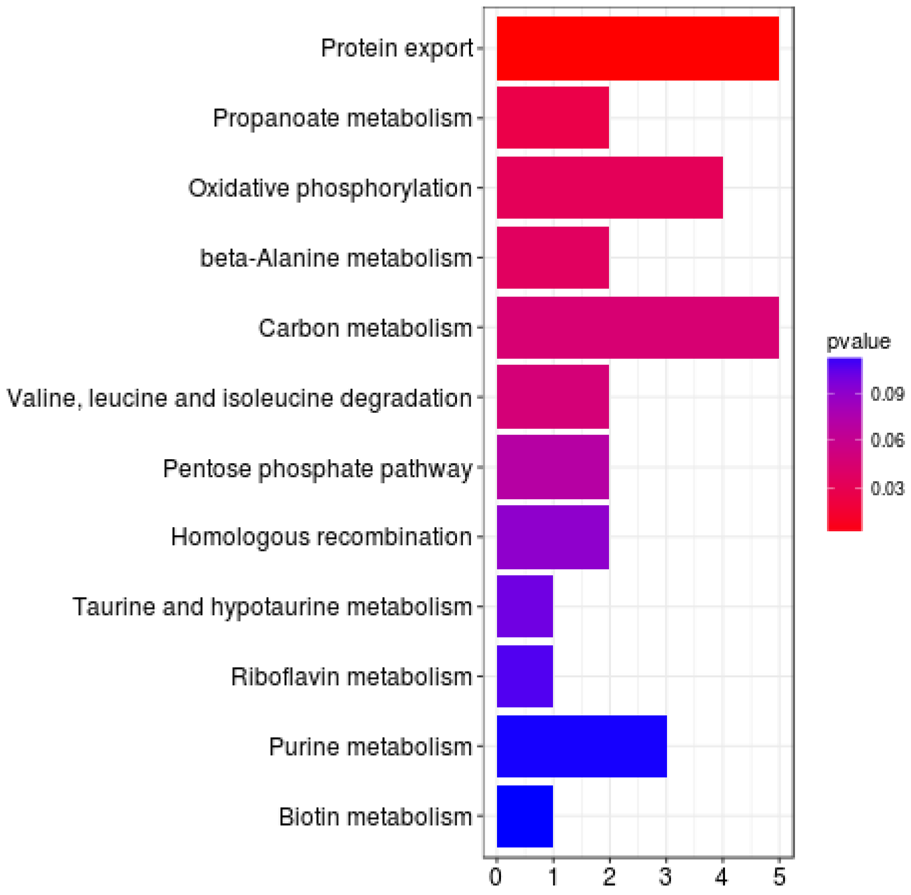

Protein export
Propanoate metabolism
Oxidative phosphorylation
beta-Alanine metabolism
Carbon metabolism
Valine, leucine and isoleucine degradation
Pentose phosphate pathway
Homologous recombination
Taurine and hypotaurine metabolism
Riboflavin metabolism
Purine metabolism
Biotin metabolism
0
1
2
3
4
5
pvalue
0.09
0.06
0.03

Table S1

Number	Gene	Unigene id	Forward primer sequence (5'–3')	Reverse primer sequence (5'–3')
1	CBL-interacting serine/threonine-protein kinase 12	cluster_contig33312	AAGATCGACATTTGGTCCTG	CAAGGATCCCGTATGATCTC
2	Calcium-binding protein CML38	TRINITY_DN43916_c0_g1_i1	CGATATTCTCCCGTCTTTGT	GAGAAAATTCTCCCGAATGG
3	Full=Glutaredoxin-C4; Short=AtGrxC4	TRINITY_DN38584_c2_g1_i3	CGCGTCTTGGATATCAACC	GATACGATCTCCTCCAGACC
4	Phototropin-1,phot1	TRINITY_DN46984_c2_g2_i3	AAAAATGTGATGCTCAACCG	CGTCCAACAGCATAAATAGC
5	Protein BIC1	cluster_contig37221	GAGCATCTGGATACCCGAC	TTGGCGCCGAGGTTGAT
6	Light-harvesting complex II protein 5,LHCB5	TRINITY_DN45074_c2_g1_i5	TTGATGGGGATGTTCTTACC	GACTACGGCTATGATCCATT
7	Putative xyloglucan endotransglucosylase/hydrolase protein 13;XTH-13	cluster_contig32082	GGCGTGATAAACAAGAATGG	CTATCTCATCATGGAAGGGC
8	Probable zinc metalloprotease EGY2	cluster_contig15073	ACCAAGCTTTATAGGGGAGT	CATGAGGTTTGGGAAATTCAG
9	Jasmonate ZIM domain-containing protein 1; Short=OsJAZ1	TRINITY_DN44883_c0_g1_i1	GAAGATCGTCAATTGTGCAG	TCATTCACAAGAACTGAGGG
10	Auxin-responsive GH3-like protein 8; Short=OsGH3-8	cluster_contig22354	TAGTGTACTCCACGATCCTT	CAACTCGACAAGGAATACGA
11	Abscisic acid 8'-hydroxylase 1; Short=ABA 8'-hydroxylase 1; AltName: Full=Cytochrome P450 707A1	cluster_contig15994	CATCGAACGCTATGGAATTG	GGGTCTATCTTCAAAAGCCA
12	*Act11*		ATGTGGCACTGGACTATGAGC	AGAGTTGTAAGTGGTTTCGTGAAT
